# How workplace learning is put into practice: contrasting the medical and nursing contexts from the perspective of teaching and learning regimes

**DOI:** 10.1007/s10459-022-10195-7

**Published:** 2022-12-02

**Authors:** Matilda Liljedahl, Erik Björck, Klara Bolander Laksov

**Affiliations:** 1 Department of Oncology, The Institute of Clinical Sciences, The Sahlgrenska Academy, University of Gothenburg, Gothenburg, Sweden; 2 Department of Oncology, Sahlgrenska University Hospital, Gothenburg, Sweden; 3 Department of Molecular Medicine and Surgery, Karolinska Institute, Stockholm, Sweden; 4 Clinical Genetics, Karolinska University Hospital, Stockholm, Sweden; 5 Department of Education, Stockholm University, Stockholm, Sweden

**Keywords:** Clinical education, Qualitative research methods, Teaching and learning regimes, Workplace learning

## Abstract

Health professions education places significant emphasis on learning in the clinical environment. While experiences of workplace learning have been extensively investigated, practices of workplace learning explored through field work have been less utilized. The theoretical framework of teaching and learning regimes acknowledges aspects of power and conflict in its consideration of what guides teachers and learners in their practice of workplace learning. This study aimed to explore practices of workplace learning in the two adjacent healthcare professions; medicine and nursing. We adopted an ethnographic qualitative design. Field observations and follow-up interviews were performed in three clinical departments and the data set comprised 12 full days of observations and 16 formal follow-up interviews. Thematic analysis was performed deductively according to the theoretical framework. Four teaching and learning regimes were found in the data. In the medical context, workplace learning was either practiced as reproduction of current practice or through stimulation of professional development. In the nursing context, workplace learning was either based on development of partnership between student and supervisor or on conditional membership in a professional community. The medical and nursing contexts demonstrated varying underpinnings and assumptions relating to teaching and learning. The respective practices of workplace learning in the medical and nursing context appear to hold substantial differences which might have implications for how we understand practices of workplace learning. We further conclude that the theoretical framework of teaching and learning regimes in this study proved useful in exploring workplace learning.

**Supplementary Information:**

The online version contains supplementary material available at 10.1007/s10459-022-10195-7.

## Introduction

Workplace learning is an essential component of health professions education (HPE) and is referred to as learning situated in a setting originally and primarily designed for practice, that is, ‘work’ (Tynjälä, 2008). There are various models, concepts and theories describing learning in general and workplace learning specifically (Bleakley, 2006; Mann, 2011). Sfard (1998) described two metaphors of learning, the acquisition metaphor and the participation metaphor, which guide the practice of learners and teachers. These metaphors have come to have significant influence on the understanding of learning in healthcare professional education. The acquisition metaphor focuses on learning as individual achievement of knowledge while the participation metaphor draws attention to the process of becoming a member in social communities. The two metaphors are not mutually exclusive but can coexist and together guide the practice of teachers and learners (Sfard, 1998). Another perspective has been added by Fuller and Unwin (2011), who have outlined the model of restrictive and expansive learning environments and argue that expansive environments are preferable as they enable newcomers to assume the status of a learner and in these environments it is thereby accepted to make time for supervision and mentoring.

Narrowing the focus to workplace learning in healthcare more specifically, Strand et al. (2015) have described three metaphors on how clinical teachers conceptualise medical students’ learning in clinical settings: learning as membership, learning as partnership and learning as ownership. Learning as membership emphasises social aspects and views learning as becoming a member of a social community. Learning as partnership stresses the relational features of workplace learning and views learning as sharing and collaborative meaning-making. Learning as ownership accentuates individual aspects, and learning is hence viewed as an acquisition of personal knowledge and skills. Even though learning as membership and partnership most naturally resides within the learning as participation-metaphor and learning as ownership relates to the acquisition-metaphor, Strand et al. (2015) point out how workplace learning to a large degree are influenced by the nature of interrelationships between learners and workplaces. It is argued that workplace learning is constructed through an interdependence between workplace affordances and individual engagement and hence shaped by both workplace norms and individuals’ agency (Billett, 2004, 2016).

These models of learning are useful in advancing our understanding of workplace learning, as they move beyond student satisfaction and instead highlight the way in which learning is constructed in work-based settings. It has recently been highlighted how research tends to neglect the impact of power dynamics in teaching and learning (Paradis & Whitehead, 2015; Stalmeijer & Varpio, 2021; van der Zwet et al., 2014) and there are arguably potential conflicts embedded in these models.

From the perspective of educational development within higher education, Trowler & Cooper (2002) have introduced the concept of teaching and learning regimes (TLRs). A TLR is understood to be a set of assumptions, rules and practices forming the basis for teaching and learning in higher education. In a TLR, implicit theories and tacit assumptions of learning guide the way teachers and students interact. It is hence implied that a TLR is primarily socially constructed. A TLR determines the circumstances under which teaching and learning might take place. Trowler & Cooper (2002) argue that teachers and learners do not act and interact in historical vacuums; they are influenced by the norms, values, practices and routines of their social community and current workplace. As such, the practices of workplace learning are guided by social rules and assumptions, which can thus be understood as being a *regime for teaching and learning*. Eight components form the basis of a TLR (described in Table 1).

**Table 1:** Overview of the core components constituting a teaching and learning regime according to Trowler and Cooper (2002)

Aspect of a TLR	Description
Identities in interaction	How individuals identify themselves in relation to others and in relation to teaching and learning. Identities are understood to be fluid, highly dependent on contextual factors and inherently relational
Power relations	How power is distributed between individuals and groups as well as how the position of teaching and learning holds compared with other orientations of an organisation
Codes of signification	Artefacts representing the underlying meaning given to, e.g. knowledge and activities
Tacit assumptions	Stereotypical images of, e.g. the nature of students and what constitutes a good supervisor. Tacit assumptions underpin practices and rules and are often taken for granted in institutions
Rules of appropriateness	Implicit principles on what students and supervisors can and cannot do according to tacit assumptions. Such rules may visualise themselves only when a new practice is introduced and immediately rejected due to inappropriateness
Recurrent practices	Habitual routines according to which individuals act. Actions and behaviours in individuals are the tacit assumptions and rules of appropriateness that are realised. Recurrent practices are understood to be socially learned and situated and are hence also resistant to change initiatives
Discursive repertoires	The frequent phrases and words used to represent ways of thinking and understanding the world. The discursive repertoires hence bring with them connotations of concepts
Implicit theories of learning and teaching	Underlying understanding of teaching and learning guiding individuals’ way of approaching a learning or teaching situation

Salient in a TLR is the assumption that there are underlying conflicts between various TLRs, which will mitigate the possibility of undertaking educational development and innovation (Trowler & Cooper, 2002). Even if various TLRs are not, by definition, irreconcilable, they are thought of as more or less compatible. As such, the coexistence of various TLRs is not surprising but, rather, anticipated. As a sociocultural theory, TLR shares some features with more familiar ones such as communities of practice (Wenger, 1998). However, a community of practice is usually portrayed as homogenous, relying on volunteering membership (Wenger & Snyder, 2000), hence implying individuals’ intentional belongingness.

In health professions education there are many potential conflicts. The most commonly referenced conflict is that occurring between different professions (Hall, 2005; Stalmeijer & Varpio, 2021), with that between medical and nursing schools and healthcare institutions (Tynjälä, 2008) and between supervisors and students (Lempp & Seale, 2004) assuming similar relevance. Paradis and Whitehead (2015) identified a lack of attention to power and conflict in the interprofessional education literature, which they suggest indicates a hesitance to engage with these issues. The framework of TLR can therefore assist in deepening our understanding of aspects such as power and conflict in the context of HPE. In medical education, Teunissen (2015) has suggested “reifications” as a level of analysis for workplace learning which shares some aspects with a TLR. Understanding the various TLRs or reifications prevailing in HPE does not only hold the potential to inform our conceptualisations on workplace learning, it can also be helpful for teachers striving to address current challenges in clinical education.

In clinical environments, students from various professions undertake clinical placements, sometimes in the very same setting. In a programme of research investigating the medical and nursing context, we have shown how medical and nursing students have different approaches to learning (Liljedahl et al., 2015) and various ways in which they interact with clinical learning environments (Liljedahl et al., 2016, 2019), indicating that there might be cultural differences in how teaching and learning were practiced in these contexts. In this study, we therefore assumed both contexts to be of interest. As previous research has primarily focused on students’ experiences and perceptions of workplace learning, we decided to here focus on how workplace learning is put into practice, hence to search for the theories-in-use (Argyris & Schön, 1974).

The aim of this study was therefore to explore practices of workplace learning in the medical and nursing contexts. The research question was posed as follows: What teaching and learning regimes can be found in the medical and nursing contexts?

## Methods

### Design

This study was performed within an interpretative paradigm, meaning that we viewed knowledge as relative and contextually constructed in interaction between researchers and the field of study (Illing, 2010). We designed an observational study in line with an ethnographic method (Atkinson & Pugsley, 2005) and thus focused on culture-sharing groups as the unit of analysis (Creswell, 2012). Each context (profession) was viewed as a ‘case’, meaning that medicine (both students and the workplace) was considered as one culture-sharing group and nursing as another.

In this study, we observed teaching and learning in action and performed follow-up interviews in clinical settings. Ethical approval was given from the Regional Ethics Committee in Stockholm and informed consent was obtained from all participants. The research team comprised various perspectives and disciplines. ML is a recently graduated medical doctor (at the time for data collection, she was a fifth-year medical student) and holds a PhD in medical education. EB is medical educator, senior consultant and associate professor in clinical genetics. KBL has background in sociology and is professor of higher education and senior researcher in medical education. So, while ML and EB are clinicians, KBL provided an outsider perspective on the clinical environment. As ML is in the beginning of her career, she could take a student perspective while EB has been a medical teacher for many years and therefore could take more of a supervisor perspective.

### Setting

The study was set in academic city hospitals in Stockholm, Sweden. The 5.5-year medical programme has a traditional curriculum with a mix of early clinical attachments. The clinical phase of student training is based on clinical rotations whereby teachers affiliated to a medical university account for theoretical education, and doctors at the hospital serve as clinical supervisors. Students’ placements within regular departments and wards are mixed with seminars, lectures and formal training in the skills training labs. Clinical rotations vary from two to twelve weeks in the same department, but placements in each ward can be shorter. Students are supervised by clinicians on duty and seldomly assigned a dedicated supervisor.

The 3-year nursing programmes are arranged so that the majority of the theoretical education is carried out at the higher education institutions, and students’ clinical placements mainly takes place at academic hospitals. During these clinical placements, nursing students are usually assigned to a specific ward or unit for two to eight weeks and supervised by one or two dedicated supervisors during the entire placement.

### Data collection

Field observations were performed at three clinical departments during a period of 18 months in 2013–2014. Site one was an orthopaedic surgery department, site two was a paediatrics department and site three was an internal medicine ward. The clinical departments were selected through a negotiation with gatekeepers, who provided access to the workplaces. One gatekeeper was a head of department, another was head of a clinical ward and the last was a clinical teacher. The medical and nursing students at each department were asked to participate in the study, with all agreeing. The included departments regularly have placements and none of the departments had a specific interprofessional curriculum.

The first author performed observations in which students were shadowed during entire shifts. The first author had no prior personal experience from the included sites. Observations were non-participatory, meaning that the observer undertook no clinical work and mainly observed practice (Hammersley & Atkinson, 2007). The observer explained the purpose of the observations to students and staff. Field notes of both descriptive and reflective character were taken and transcribed the same day or a few days following the observations. Formal follow-up interviews were held in close connection with the observations with students, supervisors and other significant individuals. The interviews were guided by an interview guide constructed by the research team (Appendix 1). Moreover, the interviewees were given the opportunity to reflect on the observed events. In addition, informal interviews and discussions were held with students and staff during the observations. In total, 12 full days of observations and 16 formal follow-up interviews (4 medical students, 5 nursing students, 3 medical supervisors, 3 nursing supervisors and 1 nurse manager) were included in the data and subject to analysis. The medical students who were shadowed were in their third, fourth or fifth year of studies while the nursing students were in their second or third year of studies.

### Data analysis

The data set has previously undergone analyses addressing other research questions and results have been published elsewhere (Liljedahl et al., 2016, 2019). Initial analyses addressing the current research question were performed iteratively with the data collection, guiding further observations and interviews. A thematic analysis was performed as a way of making sense of the data (Boyatzis, 1998). The analysis was performed deductively, meaning that the TLR framework was used as an analytical tool throughout the analysis (Braun & Clarke, 2006). The thematic analysis of this study took the following steps: (1) reading of and familiarisation with the data; (2) collaboratively searching for patterns in the data through discussions within the research team; (3) in-depth reading and analysis of the data, with the identification of meaning units corresponding to the components of a TLR; (4) grouping of meaning units into themes and (5) integrating themes into the framework of teaching and learning regimes. Throughout the entire process, the research team together reflected upon the impact of the observer on the collected data as well as researchers’ various perspectives and preunderstandings. The first author performed data collection and had the main responsibility for analysis to progress, although in close collaboration with all authors. Original field notes and interview transcript were in Swedish, and quotes used in reporting results in this paper were translated to English in late stages of manuscript preparation.

## Results

Through the analysis of practices of workplace learning, we identified two teaching and learning regimes in the medical context and two regimes in the nursing context. In Table 2, all aspects of a TLR in the four identified regimes are described. The regimes were not mutually exclusive as both individuals and sites demonstrated tendencies from several regimes.

**Table 2:** Overview of results. Practices of workplace learning were analysed according to teaching and learning regimes. An overview of the aspects in the four teaching and learning regimes are presented in the table

	Teaching and learning regimes			
	Medical context		Nursing context	
Aspect of a teaching and learning regime	Reproducing practice	Professional development	Partnership	Conditional membership
Identities in interaction	Supervisor as guide; student as visitor	Supervisor as mentor; student as mentee	Supervisor and students as partners	Supervisor as supervisor; student as student
Power relations	Supervisor as commander; student as follower	Supervisor as salesman; student as customer	Supervisor as advocate; student as protégé	Supervisor as minder; student as contender
Codes of signification	A mandatory schedule designed for exposure to variety	Taking time for dialogue	Personal learning plan as a contract for learning	Hours on duty as an artefact for learning
Tacit assumptions	Fear of students missing knowledge	Trust in the system of medical education	Establishing mutual trust and a safe environment	Assuring a minimum required level of knowledge and skills
Rules of appropriateness	Exposure to as much variety of practice as possible	Give students individually designed tasks	Knowing routines and practices gives access to learning	Adopting routines and practices gives access to the community
Recurrent practices	Imitation of demonstrated practice	Facilitation of students’ reflection	Co-protection of students’ independence in providing patient care	Monitoring students’ independence in providing patient care
Discursive repertoires	Stressing the most important thing	Acknowledging various practices and complexity	Students’ legitimacy is emphasised	Professional requirements are emphasised
Implicit theories of learning and teaching	Learning through reproduction of practice	Learning through engagement in professional development	Learning through collaboration with a partner	Learning through negotiation with the professional community

### Medical context: regime of reproducing practice

The regime of reproducing practice (Table 2) featured an ambition to educate the next generation healthcare professionals in an effective and efficient way. Supervisors adopted the role of a guide in relation to students. They gave students hints and tips about how to behave and invited them to undertake a ‘tour’ of their workplace. Students adopted the role of a visitor who interestingly followed practice with a limited degree of engagement in practice (*identities in interaction*). As students were only visitors, supervisors did not take full responsibility for students’ development and learning but rather guided them until they handed them over to the next guide. Thus, the student-supervisor relationship was a transient one. Although involving students in some activities, supervisors seemed to hold many other patient-related assignments in which they did not engage students.

The observer asks the student how the previous day turned out.

Student: *Well, it was very calm* (sounds disappointed). *We* [the supervisor and herself] *only had two patients… Or perhaps three. Because she* [the supervisor] *did some rounds by herself, I do not really know when. And then she seemed to be occupied in other places as well.* (Field note, site three)

In terms of power, supervisors were commanders of clinical practice and could mandate in which way learning was supposed to be put into practice. Students here followed the supervisors’ delimitation. In these cases, the *power relation* was clear; supervisors were in charge, and students were followers. As a *code of signification*, the schedule for clinical rotations implied an effort to expose students to a variety of fields and practices and hence, schedules were carefully designed for students to make the most of each placement.

Supervisor: *We have a morning meeting at 9 AM and then round for one to three hours depending on the patients. I will try to teach as much as possible when rounding patients. Perhaps it is good to switch teams during the week so that you can experience as much as possible.* (Field note, site three)

Present in this regime was a *tacit assumption* that students might miss out on knowledge if not exposed to it. For supervisors, this could mean that they felt the urge to teach students even if they admitted that transferring information was perhaps not the best way to learn.

The supervisor is stressed as time is running out and she has a lot of content left to share with the students.

Supervisor: *My time schedule always seems to burst. I think I talk too much.* (Field note, site one)

The dominating *rule of appropriateness* emphasised students’ observation of practice and they were not expected to contribute to medical practice in a significant way. Supervisors continually delivered guidance on procedures and administrative tasks by giving detailed instructions that students could mimic. As such, the *recurrent practice* of this regime was that students imitated the practice demonstrated by supervisors.

Supervisor (to a student): *Before your shift ends, you should always check the list of drugs. And changes in medication should be specified in the discharge note.* (Field note, site two)

Accordingly, the *discursive repertoire* included definitions of the critical mass of knowledge to learn, and students appreciated being given clear instructions and sought to learn the essential parts of the current field of practice. Supervision in this regime was about guiding, teaching and educating students as future professionals, which manifested in demonstrations which students could model and imitate. Students engaged in observations of medical doctors and used the observations as inspiration for their own development. Workplace learning was thus enacted through students’ reproduction of practice (*implicit theory of learning and teaching*).

### Medical context: regime of professional development

The regime of professional development (Table 2) included challenging students’ conceptions about the current practice and encouraging them to think outside the box. Supervisors identified themselves as student mentors, meaning that they put effort into establishing relationships built on trust. Students could respond to supervisors’ engagement by taking on the role of a mentee (*identities in interaction*). Even though the relationship could be transient in nature, at times only a few hours, it sometimes included a reciprocal engagement and commitment, thus enabling an exchange of feedback between the two.

Four students attended a three-hour seminar with a supervisor whom they had not previously met. The seminar starts out a bit rigidly, however, after the seminar, a student says:

*Things were a bit slow in the beginning, but as the day progressed, he really grew into his role as a supervisor…* (Field note, site one)

In terms of *power*, supervisors acted almost as salesmen, striving to influence student engagement in clinical practice. Students, however, sometimes took on a hostile approach, e.g. passively listening during rounds and refraining from engaging in discussions. In that way, students acted as selective customers *who deliberately choose their engagements*. In relation to teaching and learning, the power tilted towards the students.

In this regime, taking time to create a dialogue for professional development was a *code of signification*. A particular supervisor could for example encourage students to drop out of mandatory activities.

In the elevator, a student asks a supervisor whether he can join him in surgery.

Supervisor: *Of course* (cheerful)*. Only show up in the morning.*

Student: *Well, that can be a bit tricky with our busy schedule.*

Supervisor: *But it won’t kill anyone if you are away for a day.* (Field note, site one)

In terms of teaching and learning, there was a *tacit assumption* that the formal path of education assured students’ learning. Accordingly, both supervisors and students showed reliance and trust in the system of medical education. As such, supervisors dared not to insist on the specific knowledge and practices of their specialty but rather viewed their role as contributing to students’ overall progress.

*I try to hold the discussion on a higher, more theoretical level so as not to only list differential diagnoses… but to be able to relate them to each other and determine which one could be the matter in a specific situation.* […] *For the students to be able to put everything into context.* (Interview with supervisor, site two)

In line with the focus on students’ development, it was a *rule of appropriateness* to give students assignments to learn from.

*Usually, students attach value to being in the operating room as such, but such activities are useless. Sure, it can be fun to watch, but it does not contribute to this... this boring work that you actually need to learn.* (Interview with supervisor, site two)

The *recurrent practice* of this regime involved supervisors’ endeavouring to facilitate students’ reasoning. Instead of being strictly focused on the content of knowledge, supervisors admitted time for discussing a wider scope of issues with students, related to their personal and professional development. In this way, students were given the opportunity to develop their own critical thinking and questioning and were encouraged to take initiative for their own learning.

As a *discursive repertoire*, students were told that there are several possible ways of doing things. Supervision was practiced through facilitating reasoning by asking questions and requesting students to take a position in decision-making. Students responded in different ways to this approach of supervision. Some took the opportunity to develop and thus engaged in discussions around the patients. Others seemed to be intimidated and tried to dodge the situation. This regime, where workplace learning was enacted through an engagement in professional development (*implicit theory of learning and teaching*), therefore seemed to challenge the traditional role of medical students.

### Relationship between the two regimes in the medical context

In the medical context, examples of both regimes were seen across sites, specialities and stages of training. The tendency to embrace one regime or the other seemed to depend mainly on the individual supervisors’ preference and experience where more experienced supervisors practiced the professional development regime to a greater extent. For instance, Dr. X, who tended to lean against the reproducing practice regime, told the observer:

*You will see that Dr. Y and I have very different styles of teaching. Dr. Y lets the students talk more. In my seminars, I think that I talk more than them. But we run out of time otherwise, […]. But who knows what the best way is?* (Field note, site one)

Here, Dr. X was aware of the different practices in the current context and openly acknowledged the potential conflicts between regimes. As Dr. X points out, there seems to be an ongoing discussion and inquiry about how clinical medical education, in its most optimal way, can and should be carried out.

### Nursing context: regime of partnership

In the nursing context, one regime was built on a partnership (Table 2) between the supervisor and student whereby they collaborated closely to work out the challenges of patient care. The supervisors in this regime were identified as experienced colleagues, friends or even older siblings. The students were identified as novices and unknowledgeable (*identities in interaction*). Students felt comfortable with this identity as supervisors openly declared their own insecurities and shortcomings. The student-supervisor relationship was thus characterised by a sense of partnership built upon mutual recognition.

A student is about to meet and introduce herself to a newly admitted patient to the ward. Her supervisor is following her to the patient room.

Student: *I am a nursing student and will take care of you; that is ok, right?*

Patient: *Yes, I guess so.*

Supervisor: *And I am her big sister today.*

Everybody laughs together. (Field note, site one)

The power-imbalance between the student and supervisor was mitigated due to the partnership (*power relations*). In relation to other staff, or the school, supervisors here acted as advocates whose role was to protect students. Moreover, as students lacked formal power, they became the supervisors’ protégé.

A salient *code of signification* was the learning plan whereby the supervisor and student agreed on how learning was supposed to take place. These plans defined the learning outcomes and served as a contract. Underpinning the practice of teaching and learning was the *tacit assumption* that learning needed to take place in a safe environment of mutual trust between the student and supervisor.

For students, it was understood as a *rule of appropriateness* to know and familiarise themselves with the routines and practices of the workplace. Knowing the routines was a way for them to gain access to patient care and, thereby, to learning. The *recurrent practice* of the partnership regime was to let students care for patients independently. Supervisors facilitated this through an ongoing discussion regarding what students felt comfortable doing independently.

A student has prepared an infusion for a patient.

Supervisor: *Will you manage on your own?*

Student: *Yes, I think so. I think I can do it.*

Supervisor: *Yes, I know that you can.* (Field note, site two)

As such, supervisors had a protective approach towards students, meaning that they strived to provide the best possible prerequisites for student learning. A *discursive repertoire* was the emphasis on student legitimacy. Supervisors could habitually respond that they would take care of a task immediately upon noticing that a task required attention. They then corrected themselves, stating that the specific task was the responsibility of the student.

A doctor, a nursing student and her supervisor are discussing a patient.

Doctor: *Ok, let’s check myoglobin.*

Supervisor: *Sure, we will do that. Or, I mean…* (to the nursing student) *you will do that.* (Field note, site two)

Supervision was about supporting students and serving as a pillar in their learning. Students were engaged with their patients and used their patients as an inspiration, incentive, and motivation for learning. Workplace learning was in this regime implicitly understood as being facilitated through close collaboration between two partners; the supervisor and student (*implicit theories of learning and teaching*).

A nursing student has just discharged a patient.

Supervisor*: I am so proud. Look, I even get a tear* (laughs).

Student: *It is like seeing your children leave home*. (Field note, site one)

### Nursing context: regime of conditional membership

The regime of conditional membership (Table 2) was built upon an established tradition of supervising students in the clinical workplace, with pre-set roles for the actors involved. Supervisors were simply identified as supervisors and students as students (*identities in interaction*). The relationship took on a formal character whereby both actors accepted the roles and maintained a professional distance. In relation to *power*, the supervisors were minders to patient care, thereby potentially hindering student participation in practice. Here, students were contenders claiming the right to practice implied in the student role.

*Sometimes, they* [the supervisors] *do not know what I can do and what they are supposed to teach me.[…] Then I’ll need to assert my right to practice and demonstrate my knowledge and such.* (Interview with student, site three)

The *relation of power* was thus obvious; supervisors decided the extent to which students could become involved and how learning was supposed to take place. The completed hours on duty were a *code of signification*, which implied that students had learned sufficiently. Therefore, students’ year of studies had implications for their degree of involvement in patient care and more senior students were hence allowed to interact with patients to a higher degree.

*If there are first-year students here, then they are not allowed to do as much but even less.* (Interview with clinical manager, site two)

The practice of teaching and learning was underpinned by a *tacit assumption* that the minimum required level of knowledge and skills needed to be assured. Accordingly, students’ development and competence were related to the role of a professional nurse and what was expected of this individual. A central *rule of appropriateness* was for students to adopt the routines and practices of the workplace. Adopting the routines was important as this enabled access to the community of nursing.

A student brings along a note to the supervisor regarding contagion prevention (a checklist to avert nurses from exposing themselves to infections).

Supervisor: *Oh, I forgot that. And I am responsible for the entire project of contagion prevention.* (Field note, site one)

Even though students were allowed to interact independently with patients, as a *recurrent practice*, supervisors would monitor students on and off, thus ensuring that they performed their tasks correctly. In that sense, supervisors habitually oversaw students’ interactions with patients.

The distribution of responsibility was clear and, in that sense, the requirements of the professional role were emphasised as a *discursive repertoire*.

Supervisor: *As a nurse, you are constantly busy and need to juggle to make things work. What happened to the patient in room five?*

Student: *He was referred to another department.*

Supervisor: *Ok, so when will we know if he will be accepted there?*

Student: *I do not know. But he still has his intravenous catheter.*

Supervisor: *Ok, if we do not hear anything by 1.30 PM, we will have to call and ask them. And your other patient, is he alive?* (Not literately; the supervisor is wondering how the patient is doing)

Student: *Yes, I saw him in the corridor a moment ago.*

Supervisor: *Ok, so in that case, he is alive.* (Field note, site two)

Supervision was about socialising students into the nursing role, thereby including them in the community. Students engaged in social activities such as informal chats about activities outside work with the ambition of being accepted and included. Workplace learning was indirectly understood to happen through a negotiation with the professional community regarding basic values (*implicit theory of learning and teaching*) and thus enacted through a conditional membership.

### Relationship between the two regimes in the nursing context

In the nursing context, the regimes were to a large extent situated in a specific site meaning that just as natural as one regime could be in a setting or situation, as natural could the other regime be in another setting. The educational approach and strategy in a clinical placement, or for a specific supervisor or student, seemed fixed and therefore potential conflicts between the regimes were not openly discussed.

## Discussion

The clinical environment is usually highly valued by students as an important setting for learning. The way in which workplace learning is enacted in clinical environments is understood to be guided by conceptualisations of teaching and learning and based on the history, traditions, norms and values of workplaces (Billett, 2004, 2016). Through employing the framework of teaching and learning regimes (Trowler & Cooper, 2002) we have investigated contexts of medicine and nursing and described practices of workplace learning.

Workplace learning in the medical context was dominated by a notion that learning was an individual act, either through professional development facilitated by supervisors or through acquiring knowledge transferred from a supervisor (Table 2) the latter is a focus which can be traced back as far back as the master-apprenticeship model. In the medical context, role models are known to be of high influence (Bleakley & Bligh, 2008; Liljedahl et al., 2015). Imitating and observing role models is a well-established educational strategy in the medical community (Lieff & Albert, 2012; Skipper et al., 2016). Exploring the development of medical teachers from a community of practice perspective, Cantillon and colleagues found clinical communities of practice to be highly conservative and that there was a tendency to reproduce practice (Cantillon et al., 2016). Likewise, Teunissen (2015) highlights recurrent practices as a feature of medical practice whereby norms, values, priorities and practices can continue despite the fact that individuals come and go. Based on previous and present research, we believe that the notion of reproducing practice is deeply embedded in the culture of medicine. By contrast, the regime of professional development seemed to endeavour into creating what van der Zwet et al. (2011) called a ‘developmental space’ for students; thereby allowing learning to be undertaken in a more reflective manner.

Workplace learning in the nursing context was, in this study, dominated by a relational approach (Table 2), meaning that supervisors and students needed to manage social relationships to gain access to practice and learning. This is well aligned with previous research in which nursing students emphasised belongingness to the community of nursing as a prerequisite for learning (Levett-Jones & Lathlean, 2008). Further, students have advocated that mastering the relationship with staff is essential for learning (Levett‐Jones et al., 2009). In a previous study, it was found that nursing students did not necessarily desire to belong. In that study, students demonstrated a hesitance to being included in the nursing community as they did not agree with some of the basic values of the profession (Liljedahl et al., 2016). In the partnership regime described here, students and supervisors seemed to interact on more equal terms than in the community regime whereby the latter, by contrast, appeared to implicitly ask students to join the community without negotiating the norms and values of the community. While the regime of conditional membership reminded of a restrictive learning environment, the partnership regime, by contrast, resembled an expansive learning environment (Fuller & Unwin, 2011). In terms of belongingness, students might find it easier to accept belonging in the partnership regime as they are given the possibility to influence the terms under which they join a social practice. In the conditional membership regime, however, students might instead show resistance as their role seemed to be pre-set.

Results suggest that the two contexts investigated in this study (medicine and nursing) featured varied conceptualisations of learning. While the nursing context focused on learning as a process (repeatedly and openly negotiating how learning was supposed to take place), the medical context was more concerned with the outcome of learning (continually discussing what a medical doctor should know and how they should act). In comparison with the aforementioned metaphors of learning conceptualised by clinical teachers (Strand et al., 2015), the medical context in this study seemed to have enacted more of learning-as-ownership whereas the nursing context endorsed features of learning-as-partnership and learning-as-membership. Consequently, the nursing context seemed to have adopted more of the participation metaphor of learning while the medical context seemed to have embraced the acquisition metaphor to a greater extent (Sfard, 1998). This is important, not least as power dynamics between professions might be underestimated in HPE (Paradis & Whitehead, 2015; Stalmeijer & Varpio, 2021). Based on the current and recent research, it seems pivotal to acknowledge practices of teaching and learning as influential both with-in and between professions (Cantillon et al., 2022).

A TLR is understood to be closely linked to the disciplines’ ontological and epistemological assumptions (Trowler & Cooper, 2002), that is, the worldview and the understanding of knowledge. The way in which knowledge is understood within a profession will thus affect the practice of learning in that discipline (e.g. medicine). Therefore, it is not surprising that the professions of medicine and nursing demonstrated such varying teaching and learning regimes, as the two professions stem from different traditions (Hall, 2005). Importantly, Sfard (1998) reminds us that none of the metaphors of learning alone should be translated and used in educational practice. Instead, Sfard argues for a combination of the acquisition and participation metaphor to meet individual and contextual needs. The present study therefore urges us, that rather than viewing one context as being in favour of the other, the medical and nursing context might have something to learn from each other.

In effect, we found the TLR framework beneficial for investigating dynamics of power in the clinical environment. The eight components of a TLR served as a language in which we could describe practices of workplace learning. As TLR brought forward aspects such as distribution of power and rules of appropriateness, it enabled teaching and learning not to be viewed solely as a cheerful and harmonious activity. This study therefore suggests the TLR framework to be suitable for analysing and investigating workplace learning in HPE and that it therefore should be included as a complementary workplace learning theory in the field.

### Methodological considerations

This study was limited to three departments, and therefore, future studies will have to address the transferability of the results to other settings. We consider it a strength that both a medical and a nursing context were included in this study, which enabled contrasts to be drawn between the two professions. As the observer was familiar with the clinical context, she could read silent codes but might have missed other relevant aspects she might take for granted. This likely influenced the data collection and analysis in this study and the research team was helpful in challenging ML with alternative perspectives. Analysis of the data was deductive in nature, which could provide more detailed descriptions of a few aspects in the data, with the loss of a rich overall description (Braun & Clarke, 2006). In this study, the unit of analysis was the profession as a whole, including both students and supervisors. There might be potential variations in conceptualisations between students and supervisors, and future studies could therefore focus on a single group to delineate differences.

## Conclusions

In conclusion, this study identified four teaching and learning regimes in the medical and nursing contexts which showed diverse underpinnings and assumptions in relation to teaching and learning and consequently, the medical and nursing contexts seem to put workplace learning into practice in different ways. Results in this study might therefore be used by teachers and learners to engage in a mutual discussion regarding the assumptions guiding how workplace learning is put into practice in the clinical environment. Further, we conclude that the framework of teaching and learning regimes was assistive in acknowledging power dynamics in workplace learning and this study therefore suggest TLRs to be included as a complementary workplace learning theory in HPE.

## Supplementary Information

Below is the link to the electronic supplementary material.

Supplementary file1 (DOCX 20 kb)
